# Pharmacokinetics of Dexamethasone when Administered with Fosaprepitant for Chemotherapy-Induced Nausea and Vomiting and Differences in Dose-Dependent Antiemetic Effects

**DOI:** 10.31557/APJCP.2021.22.3.871

**Published:** 2021-03

**Authors:** Fukutaro Shimamoto, Masahiro Goto, Tetsuji Terazawa, Ken Asaishi, Takahiro Miyamoto, Kazuhide Higuchi

**Affiliations:** 1 *Cancer Chemotherapy Center, Osaka Medical College Hospital, 2-7, Daigaku-machi, Takatsuki, Osaka 569-8686, Japan.*; 2 *Second Department of Internal Medicine, Osaka Medical College, 2-7, Daigaku-machi, Takatsuki, Osaka 569-8686, Japan. *

**Keywords:** CYP3A4, dexamethasone, fosaprepitant, highly emetogenic chemotherapy, pharmacokinetics

## Abstract

**Background:**

Fosaprepitant, an NK_1_ receptor antagonist, inhibits and induces cytochrome P450 3A4 (CYP3A4) as its substrate. Contrarily dexamethasone is metabolized by CYP3A4. Therefore, in combination therapy wherein both agents interact with each other, it is recommended that the dexamethasone dose be reduced in the first two days. Thus far, there are only a few studies on the optimum dose of dexamethasone after day 3. Thus, we aimed to determine the pharmacokinetics of dexamethasone on day3 when administered together with fosaprepitant and investigate the dose-dependent differences in its antiemetic effect in patients with cancer.

**Methods:**

Twelve patients with esophageal, stomach, or lung cancer received primary highly emetogenic chemotherapy (HEC). We intravenously administered 9.9 mg and 6.6 mg of dexamethasone on days 1 and 2, respectively, and 6.6 mg or 13.2 mg on day 3 together with the administration of 150 mg fosaprepitant and 0.75 mg palonosetron. We assessed the pharmacokinetics of dexamethasone on day 3 by dose and examined the dose-dependent antiemetic effect.

**Results:**

No differences were observed in the time-to-maximum concentration and blood half-life of dexamethasone between patient groups that received dexamethasone at doses of 6.6 mg and 13.2 mg. In contrast, the area under the blood concentration-time curve and the maximum concentration of dexamethasone correlated with its dose. Moreover, the blood dexamethasone concentration on day 3 increased by twofold after the administration of a higher dose than after a lower dose. The severity of nausea in the delayed phase significantly decreased in a dose-dependent manner.

**Conclusion:**

Administration of a higher dexamethasone dose on day 3 improved the antiemetic effect of the combined regimen in patients with cancer who underwent HEC.

## Introduction

Prevention of chemotherapy-induced nausea and vomiting (CINV) is essential to maintain the intensity and maximize the effect of anticancer pharmacotherapy. To undergo appropriate antiemetic treatment, the American Society of Clinical Oncology (ASCO) (Hesketh et al., 2020), National Comprehensive Cancer Network (NCCN) (National Comprehensive Cancer Network), and the Multinational Association of Supportive Care in Cancer (MASCC)/European Society for Medical Oncology (ESMO) (Roila et al., 2016) have prepared and published guidelines. The Japanese Society of Clinical Oncology Clinical Practice Guidelines 2015 for Antiemesis (Takeuchi et al., 2016) have also been published. These guidelines recommend antiemetic treatment corresponding to the emetogenic risks and recommend triple therapy with an NK_1_ receptor antagonist, a 5-HT_3_ receptor antagonist and dexamethasone as one of the antiemetic options for highly emetogenic chemotherapy (HEC).

Fosaprepitant meglumine (fosaprepitant), an NK1 receptor antagonist, was shown to be effective for treating nausea and vomiting in both the acute and delayed phases of chemotherapy infusions with a single injection (Grunberg et al., 2011). Fosaprepitant, which is a substrate of cytochrome P450 3A4 (CYP3A4), shows mild to moderate inhibitory and inducing effects on this enzyme (Sanchez et al., 2004). Contrarily, dexamethasone, a well-known antiemetic, is metabolized by CYP3A4; therefore, after combined administration of fosaprepitant and dexamethasone, it is necessary to consider any interaction between these two drugs. A pharmacokinetic study on the combined use of fosaprepitant and dexamethasone in normal healthy participants demonstrated that fosaprepitant increased the area under the blood concentration-time curve of dexamethasone by twofold on days 1 and 2; however, interaction between the two drugs was barely observed on day 3 (Marbury et al., 2011). Accordingly, a dose reduction of dexamethasone was recommended on days 1 and 2 in the above-mentioned guidelines after considering drug interaction. However, there is no consensus on the optimum dose of dexamethasone after day 3 because of insufficient studies at this time point (Hesketh et al., 2020; National Comprehensive Cancer Network; Roila et al., 2016; Takeuchi et al., 2016).

Thus, we planned a clinical study to determine the optimum dose of dexamethasone on day 3 when used in combination with fosaprepitant. We expected that CINV was suppressed in proportion to the plasma concentration of dexamethasone. We aimed to evaluate the pharmacokinetics of dexamethasone on day 3 after its combined administration with fosaprepitant and to examine whether any suppressive effect on CINV differed according to the dexamethasone dose. This study was registered with the UMIN Clinical Trials Registry (UMIN 000013782).

## Materials and Methods

*Patients*

Study participants included patients with cancer aged 20 years and older, who provided us with informed consent, had an Eastern Cooperative Oncology Group performance status of 0-2 and were treated with HEC for the first time. Patients who met the following criteria were excluded: treatment with pimozide, serious hepatic or renal impairment, a history of treatment with a regimen containing a moderate emetogenic anticancer agent; and development of nausea/vomiting within 24 hours before the initiation of chemotherapy. Patients who received multiple-day chemotherapy, including highly emetogenic anticancer agents, were eligible. The number of participants was set to 12 because a similar number patients (11 or 12) was used in studies conducted in other countries worldwide on the pharmacokinetics of dexamethasone after its combined administration with fosaprepitant in healthy individuals (McCrea et al., 2003; Marbury et al., 2011).

*Study design*

**Figure 1:**
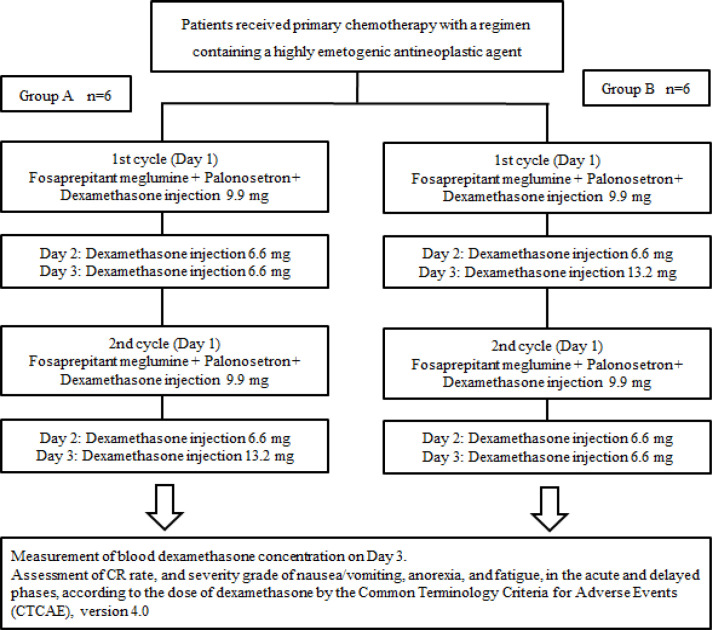
Flowchart of the Trial Method of This Study as a Randomized Open-Label Cross-Over Controlled Study. With the administration of 150 mg of fosaprepitant and 0.75 mg of palonosetron, patients in Group A intravenously received dexamethasone doses of 9.9, 6.6, and 6.6 mg on days 1, 2, and 3, respectively, in the first cycle and dexamethasone doses of 9.9, 6.6, and 13.2 mg on days 1, 2, and 3, respectively, in the second cycle. In contrast, patients in Group B intravenously received dexamethasone doses of 9.9, 6.6, and 13.2 mg on days 1, 2, and 3, respectively, in the first cycle and dexamethasone doses of 9.9, 6.6, and 6.6 mg on days 1, 2, and 3, respectively, in the second cycle

This study was a randomized open-label cross-over controlled study that was conducted at Osaka Medical College Hospital between March 2014 through October 2015, after approval by the Osaka Medical College Ethics Committee. The trial design is shown in Figure 1. Randomization was performed using the numbered container method. With the administration of 150 mg fosaprepitant and 0.75 mg palonosetron, patients in Group A also received dexamethasone doses of 9.9, 6.6, and 6.6 mg intravenously on days 1, 2, and 3, respectively, in the first cycle, and dexamethasone doses of 9.9, 6.6, and 13.2 mg on days 1, 2, and 3, respectively, in the second cycle. In comparison, patients in Group B received dexamethasone doses of 9.9, 6.6, and 13.2 mg intravenously on days 1, 2, and 3, respectively, in the first cycle and dexamethasone doses of 9.9, 6.6, and 6.6 mg on days 1, 2, and 3, respectively, in the second cycle. The protocol treatment was performed regardless of the type of anticancer agent used and the administration period. The change in the dose of antineoplastic agents between the first and second cycles was not considered to be a protocol deviation.

*Data collection*

Blood samples (5 mL) were collected into EDTA-container tubes from patients in Groups A and B before, and 1, 4, 8, 12, and 24 h after the administration of dexamethasone on day 3 in the first and second cycles. The blood samples were centrifuged at 3,000 rpm for 10 min at 5°C. After centrifugation, the plasma was collected in cryotubes and stored in a frozen state at -20°C until analysis. The plasma dexamethasone concentration was analyzed using LSI Medience Corporation (Tokyo, Japan) with liquid chromatography-mass spectrometry. All patients in Groups A and B were requested to keep patient diaries throughout the treatment period to assess the severity of nausea, vomiting, anorexia, and fatigue.

*Assessments*

The primary endpoint of this study was to evaluate plasma dexamethasone concentrations according to its dose on day 3 of treatment. The pharmacokinetic parameters of dexamethasone, the area under the concentration-time curve (AUC_0-24h_), maximum concentration (C_max_), time-to-maximum concentration (T_max_), and blood half-life (T_1/2_) were calculated. As secondary endpoints, complete response (CR) rate (absence of vomiting; rescue treatment was defined as a complete response) and severity of nausea, vomiting, anorexia, and fatigue were assessed in the acute and delayed phases from records of patients’ diaries according to the Common Terminology Criteria for Adverse Event (CTCAE), version 4.0. Acute phase was defined as 0–24 h after the administration of highly emetogenic anticancer agents, while the delayed phase was defined as 24–120 h after the administration of highly emetogenic anticancer agents even with multiple-day chemotherapy.

*Statistical analysis*

The pharmacokinetic parameters of dexamethasone were determined by non-compartmental methods using Excel 2016. C_max_ and T_max_ were determined directly from the raw data. The elimination rate constant λ was determined by linear regression of the natural log-transformed values of concentrations in the elimination phase, and T_1/2_ was calculated as ln(2)/λ. AUC_0-24h_ was calculated using the linear trapezoidal rule. The difference in pharmacokinetic parameters between the two groups was examined using a paired t-test. Differences in the CR rate and the severity of nausea, vomiting, anorexia, and fatigue in the acute and late phases were examined using the chi-square test. All analyses were performed using JMP Pro 11 software (SAS Institute Inc., Cary, NC, USA).

## Results

*Patient characteristics*

Twelve patients with cancer who were receiving HEC for the first time and consented to participate in this study were randomly assigned to the two groups (six patients in each group). Table 1 shows patients’ characteristics. All patients completed two cycles of treatment with no protocol deviation, although one patient who underwent PI therapy required a 20% dose reduction of CPT-11 and CDDP due to the occurrence of diarrhea and fatigue.

*Pharmacokinetics of dexamethasone*

**Figure 2:**
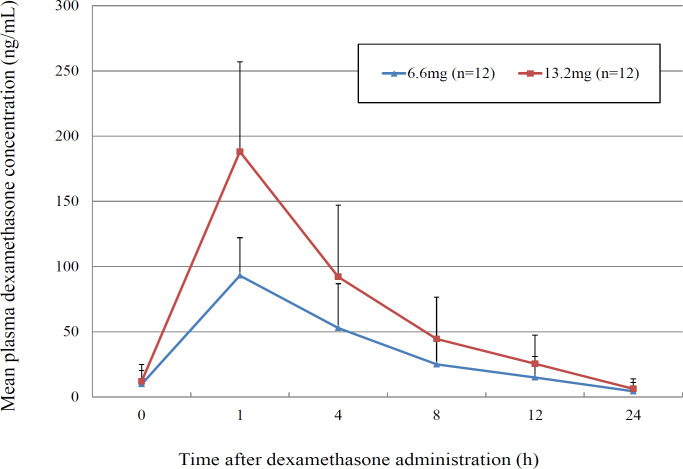
Mean Plasma Concentration Profiles of Dexamethasone Administered at Doses of 6.6 mg or 13.2 mg on Day 3, respectively, after Combined Intravenous Administration of 150 mg Fosaprepitant and 0.75 mg Palonosetron. Error bars show the standard deviation

Figure 2 shows the time course change in blood dexamethasone concentrations on day 3 of administration according to dose. Table 2 shows the pharmacokinetic parameters of dexamethasone. Almost the same time course change was observed after the administration of dexamethasone at both doses (6.6 mg and 13.2 mg); however, significant differences were not noted in T_max_ and T_1/2_ between both groups. In contrast, the blood dexamethasone concentration increased by two-fold after the administration of 13.2 mg dexamethasone compared to that with the administration of 6.6 mg dexamethasone. The AUC_0-24 h_ was 621 ng*h/mL (95% CI: 362–879) and 1261 ng*h/mL (95% CI: 735–1787), and the C_max_ was 93.1 ng/mL (95% CI: 74.6–111.6) and 188.1 ng/mL (95% CI: 144.3–231.9) after the administration of dexamethasone at dose of 6.6 mg and 13.2 mg, respectively.

*Efficacy*

**Figure 3:**
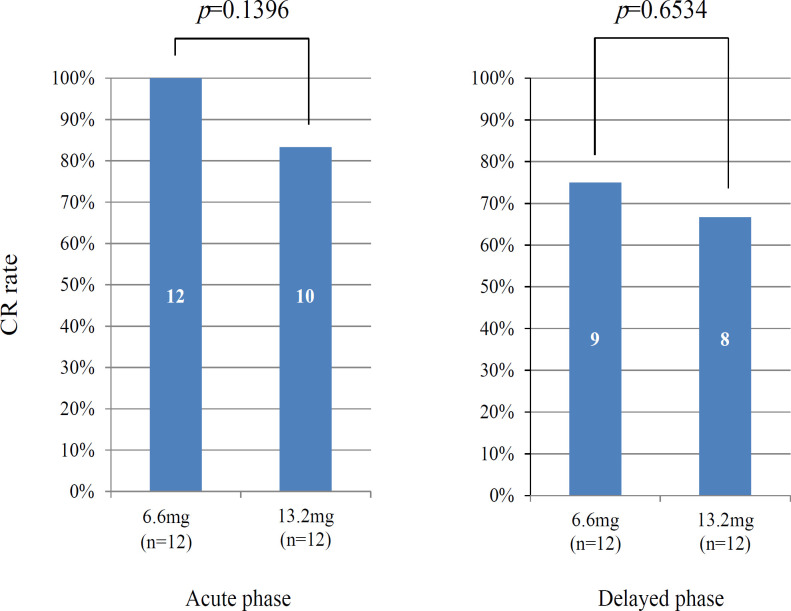
Proportion of Patients Achieving Complete Response (CR; a condition involving cessation of vomiting, rescue treatment) in the acute (within 24 hours) and delayed (25–120 hours) phases according to the dexamethasone dose. Numbers in columns represent patient numbers

**Figure 4:**
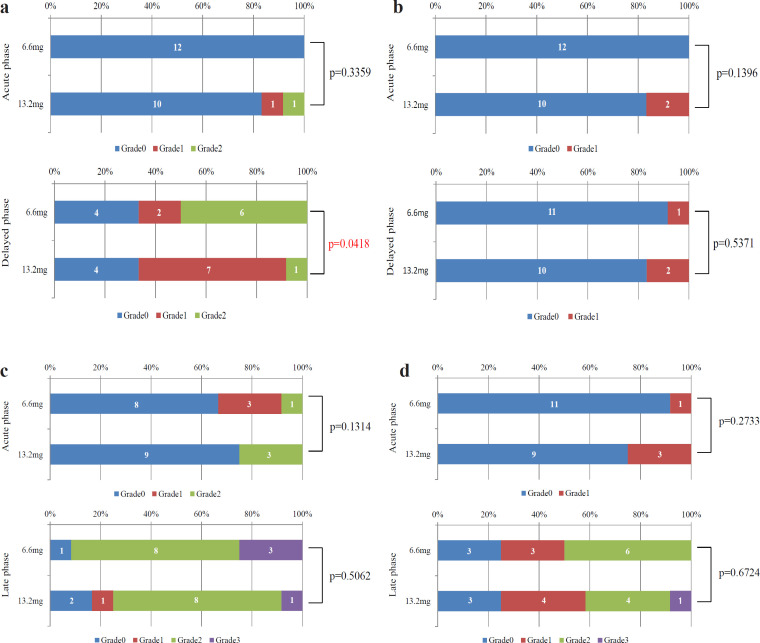
Severity Grades of Nausea (a), vomiting (b), anorexia (c), and fatigue (d), based on the Common Terminology Criteria Adverse Events in the acute and delayed phases according to the dexamethasone dose. Numbers in columns represent patient numbers

Significant differences in CR rates between the two groups were not noted. The acute phase CR rates were 100% and 83.3% (p = 0.1396), and the delayed phase CR rates were 75% and 66.7% (p = 0.6534) after the administration of dexamethasone doses of 6.6 mg and 13.2 mg, respectively (Figure 3). Figure 4a, 4b, 4c, and 4d show the distribution of the severity grades of nausea, vomiting, anorexia, and fatigue, respectively, according to the dexamethasone dose. The severity grade of nausea in the late phase according to the CTCAE was significantly lower in the group treated with 13.2 mg dexamethasone than in the group treated with 6.6 mg dexamethasone. Significant differences were not observed for the severity grade of vomiting, anorexia, or fatigue between the two groups. Serious adverse events caused by the administration of dexamethasone did not occur throughout the study.

**Table 1:** Patients’ Characteristics (n=12)

Age	Median	69 (57–76)
Sex	Male/Female	11/1
Primary cancer	Esophagus/Stomach/Lung	10/1/1
Regimen*	Preoperative CF therapy	3
	CF-RT therapy	4
	CF therapy	2
	SP therapy	1
	PI therapy	1
	DCF therapy	1
Performance status	0 / 1	5/7
Risk factors**	0 / 1 / 2	4/7/1

*Preoperative CF therapy: 5FU at 800 mg/m^2^ on days 1-5, CDDP at 80 mg/m^2^ on day 1; CF-RT therapy: 5FU at 700 mg/m2 on days 1-4 and 29-32, CDDP at 70 mg/m^2^ on days 1 and 29, concurrent radiotherapy 60 Gy/30 Fr; CF therapy: 5FU at 800 mg/m^2^ on days 1-5, CDDP at 80 mg/m^2^ on day 1; SP therapy: S-1 at 80 mg/m^2^ on days 1-21, CDDP at 60 mg/m^2^ on day 8; PI therapy: CDDP at 60 mg/m^2^ on day 1, CPT-11 at 60 mg/m^2^ on days 1, 8, and 15; DCF therapy: DOC at 30 mg/m^2^ on days 1 and 15, CDDP at 80 mg/m^2^ on day 1, 5FU at 800 mg/m^2^ on days 1-5; **Female, 50 years old or younger, alcohol intake (-), motion sickness (+), morning sickness in pregnancy (+), anxiety (+)

CR, complete response was defined as the cessation of vomiting; rescue treatment.

**Table 2:** Pharmacokinetic Parameters of Dexamethasone by Dose

	Dose of dexamethasone		*P-value*
	6.6 mg (n=12)	13.2 mg (n=12)	
AUC_0-24 h_ (ng.h/mL)[95% CI]	621 [362-879]	1261 [735-1787]	<0.01
C_max_ (ng/mL)[95% CI]	93.1 [74.6-111.6]	188.1 [144.3-231.9]	<0.01
T_max_ (h)[95% CI]	1.3 [0.7-1.9]	1.0 [0.8-1.3]	0.34
T_1/2_ (h)[95% CI]	4.6 [2.9-6.3]	4.7 [2.9-6.5]	0.87

AUC, area under the curve; CI, confidence interval

## Discussion

The present study showed that there was no difference in the time-to-maximum concentration and blood half-life of dexamethasone on day 3 between the two patient groups (dexamethasone 6.6 mg and 13.2 mg). However, the blood dexamethasone concentration on day 3 increased by twofold after the administration of a higher dose than that after the administration of a lower dose. In the delayed phase, the severity of nausea significantly decreased in a dose-dependent manner.

Delayed phase CINV in patients treated with HEC has been a major residual problem after the development of 5-HT_3_ receptor antagonists. The treatment of CINV has greatly advanced with the development of palonosetron, a second-generation 5HT_3_ receptor antagonist, and NK_1_ receptor antagonists such as aprepitant and fosaprepitant. Fosaprepitant is a water-soluble phosphorylated prodrug of aprepitant that is rapidly metabolized to aprepitant by phosphatases after its intravenous administration (Hale et al., 2000). In addition, a single administration of fosaprepitant was shown to have the equivalent efficacy of a 3-day-consecutive administration of aprepitant (Grunberg et al., 2011). Because fosaprepitant is convenient and can be administered to patients who cannot take drugs orally, its combined administration with a 5-HT_3_ receptor antagonist and dexamethasone is a standard antiemetic treatment for patients treated with HEC. However, to date, no consensus has been reached on the optimal dose of dexamethasone to be used for combined administration with fosaprepitant inside and outside of Japan because of the lack of sufficient evidence (Hesketh et al.; National Comprehensive Cancer Network; Roila et al., 2016; Takeuchi et al., 2016). 

Because fosaprepitant is a substrate of CYP3A4 and has mild to moderate inhibitory and inducing effects, any drug interaction between fosaprepitant and dexamethasone should be noted because the latter is also metabolized by CYP3A4 (Sanchez et al., 2004). A pharmacokinetic study on the combined administration of fosaprepitant and dexamethasone in normal healthy individuals demonstrated that fosaprepitant increased the AUC of dexamethasone by twofold on days 1 and 2. However, drug interaction was barely observed on day 3 (Marbury et al., 2011). Accordingly, the dose of dexamethasone was reduced on days 1 and 2 in a global phase III trial of fosaprepitant conducted outside of Japan (Grunberg et al., 2011), where dexamethasone doses of 12, 8, 16 and 16 mg were orally administered on days 1, 2, 3, and 4, respectively. However, in a phase III trial in Japan (Saito et al., 2013), regarding the combined administration of fosaprepitant and dexamethasone, the latter was administered at doses of 10, 4, and 8 mg on days 1, 2, and 3, respectively. Thus, the dose was reduced on days 1 and 2, as in the above-mentioned global phase III trial; however, the dose used was lower in the Japanese trial than in the global trial. When comparing the results of these trials, the CR rate and the severity grade of nausea in the delayed phase tended to be lower in the global trial, in which the dose of dexamethasone used was higher than that used in the Japanese trial (Grunberg et al., 2011; Saito et al., 2013). Although care should be taken when comparing the results of trials conducted in different populations, we thought the difference in the suppressive effect on nausea and vomiting observed for different dexamethasone doses was worth verifying and, therefore, planned the present study.

In this study, we focused on the dexamethasone dose to be administered on day 3, in which fosaprepitant had less influence on the metabolism of dexamethasone through its effect on CYP3A4. We assessed the pharmacokinetics of dexamethasone when administered in combination with fosaprepitant and examined whether differences in antiemetic effects occurred in a dose-dependent manner. Taking advantage of the convenience of an intravenous injectable fosaprepitant, dexamethasone was also intravenously administered. In addition, because a large volume of supplementation fluid was generally infused for approximately 3 days to protect renal function during the administration of cisplatin, dexamethasone was also administered for 3 days in this study.

Regarding the pharmacokinetics of dexamethasone on day 3, the time course changes in the blood dexamethasone concentration were almost the same between groups treated with dexamethasone doses of 6.6 and 13.2 mg. Significant differences were not observed in T_max_ and T_1/2_ between the two groups. In contrast, the C_max_ and AUC_0-24 h_ of dexamethasone increased by approximately twofold in the group treated at a dose of 13.2 mg compared to those of the group treated with 6.6 mg in proportion to the dose, indicating that the blood dexamethasone concentration increased in a dose-dependent manner. Although the assessment of the antiemetic effect of the combined administration of dexamethasone and fosaprepitant was exploratory, the results demonstrated that the severity grade of nausea was significantly lower in the group treated with a higher dose of dexamethasone than in the group treated with a lower dose. In addition, the severity grade of anorexia tended to be lower in the group treated with a higher dose of dexamethasone than in the group treated with a lower dose, although the difference was not significant.

However, this study has some limitations regarding the antiemetic effect. First, the primary endpoint of this study was the pharmacokinetics of dexamethasone when administered in combination with fosaprepitant; the sample size in this study was too small to discuss the antiemetic effect. In addition, currently available guidelines recommend the administration of dexamethasone for 4 days with an NK_1_ receptor antagonist, a 5-HT_3_ receptor antagonist; the administration period of dexamethasone is also controversial.

Our study showed that the administration of a higher dexamethasone dose on day 3 improved the antiemetic effect of the combination regimen in patients with cancer who underwent HEC. We intend to assess the antiemetic effect and the safety of combination therapy with fosaprepitant, palonosetron, and dexamethasone in a larger number of patients with cancer.

## Author Contribution Statement

All authors contributed to all processes of this clinical trial and approved the final manuscript. Fukutaro Shimamoto launched this research, analyzed the data, and wrote this manuscript. Masahiro Goto and Kazuhide Higuchi provided constructive advice when writing the manuscript.
